# Ageing is associated with exaggerated overstaying in foraging behaviour

**DOI:** 10.1038/s41514-025-00240-1

**Published:** 2025-05-30

**Authors:** Noham Wolpe, Daniel N. Scott, Mordechai L. Salomon, Matthew R. Nassar, Paul C. Fletcher, Emilio Fernandez-Egea

**Affiliations:** 1 Department of Physical Therapy, Stanley Steyer School of Health Professions, Gray Faculty of Medical & Health Sciences, Tel Aviv University, Tel Aviv, Israel; 2 Sagol School of Neuroscience, Tel Aviv University, Tel Aviv, Israel; 3 Department of Psychiatry, University of Cambridge, Cambridge, UK; 4 Department of Neuroscience, Brown University, Providence, RI USA; 5 Carney Institute for Brain Science, Brown University, Providence, RI USA; 6 Cambridgeshire and Peterborough NHS Foundation Trust, Cambridge, UK

**Keywords:** Cognitive ageing, Ageing

## Abstract

People constantly decide how much time to invest in rewarding activities. Foraging tasks assess this decision-making by measuring when individuals switch between contexts. People typically perform suboptimally in these tasks, largely due to overstaying, but it remains unclear whether this tendency changes with age independently of cognitive abilities and mental health factors. Previous research showing increased sensitivity to the opportunity cost of time in older adults predicts less overstaying, whereas a hypothesised shift towards exploitative behaviour predicts more overstaying. In an online foraging task, 350 young and older adults decided when to switch between contexts with varying reward conditions. We also assessed cognitive performance and self-reported motivation and depression. Participants consistently overstayed, and this behaviour was strongly associated with sensitivity to reward changes. Despite this, older adults selectively overstayed more without increased reward-based adaptation. Our findings show ageing is associated with exaggerated overstaying, supporting increased exploitative behaviour in old age.

## Introduction

When faced with diminishing returns from a current course of action, people must constantly evaluate whether to persist or switch to a potentially better alternative. This fundamental decision-making challenge is captured by foraging behaviour, which provides a theoretical and an experimental framework for understanding how organisms optimise resource acquisition over time. Foraging paradigms have recently been applied to examine individual differences in neuropsychiatric conditions^1,2^. However, their relevance to cognitive ageing remains underexplored, despite the well-documented changes in motivation, cognitive control and learning with age—all of which are central to decisions about persistence and switching.

The Marginal Value Theorem (MVT) provides a normative model of when to switch in foraging behaviour^3^. According to the MVT, the optimal strategy is to adopt a constant ‘patch’ exit threshold, leaving a patch when it’s marginal return falls to the average reward rate available in the environment. Such a strategy requires continuous monitoring and integration of dynamic information about background (environmental) and foreground (current patch) reward rates. The widespread age-related changes in cognition^4,5^ and brain function relevant to reward processing^6,7^ may impact these mechanisms. Thus, applying foraging paradigms to study ageing offers a way to examine how such cognitive changes may alter decisions about persistence and switching.

Although normative models suggest threshold-based decision rules, empirical evidence shows that humans often deviate from these rules, persisting by extracting diminishing resources longer than optimal^8–10^. Such ‘overstaying’ may reflect uncertainty about the environment, leading to an adaptive discounting of future reward and, hence, overstaying^8,11^, Importantly, the use of such strategies may shift across the lifespan. According to the ‘exploitative mental mode’ hypothesis^12^, ageing is accompanied by a broad shift from exploration to exploitation, which reflects cumulative prior knowledge, reduced cognitive flexibility, and changes in reward and affective valuation systems^12^. The exploitative mental mode hypothesis would thus predict increased overstaying behaviour in older adults.

However, findings on age-related changes in reward valuation are mixed^7^, and are dependent on the specific context of the decision. For example, some evidence suggests that older adults increase cognitive effort investment under high opportunity costs—that is, when the environmental rewards missed while staying in the current context are increased^13^. Higher sensitivity to opportunity costs would predict reduced overstaying in older adults. In this study, we tested these competing hypotheses, by investigating foraging behaviour in older vs. young adults.

Understanding age-related changes in foraging also requires accounting for individual differences in cognitive capacity and affective symptoms, mainly depressive symptoms^2^ and motivation^10^. Cognitive performance, particularly in terms of executive control, is known to decline with age^14^ and may also interact with foraging behaviour in old age. Similarly, depressive and motivational symptoms can both reduce goal-directed behaviour through distinct mechanisms^15^, which may in turn influence exploration-exploitation behaviour in old age^12^. These variables are crucial to disentangle normative age-related shifts from trait-level individual differences.

We examined how suboptimality in foraging behaviour, and particularly overstaying behaviours, vary across the adult lifespan. We introduced a novel online foraging task designed to reveal deviations from normative threshold behaviour, and task factors that elicit these deviations. We hypothesised that most participants would show systematic overstaying, consistent with prior work. Importantly, however, we tested whether older adults show more overstaying behaviour, consistent with the exploitative mental mode hypothesis^12^, or less overstaying behaviour consistent with heightened sensitivity to the opportunity cost of time in some contexts^7,13^. While testing these key predictions, by measuring cognitive, depressive and motivational variables, we sought to distinguish age-related strategic shifts from general individual differences.

## Results

### Condition differentiation and suboptimality

In order to understand how people should ideally behave in the foraging task, we first computed the optimal policy using optimisation methods (see Methods). We found that optimal policies depended both on the initial reward rate and the speed of decay, with the high reward rate-slow decay condition (High-Slow) prescribing the maximal stay duration 7.5 s and the low reward rate-fast decay condition (Low-Fast) prescribing the minimal stay duration of 2.05 s. The two intermediate conditions had similar optimal policies, with 4.1 s for Low-Slow and 3.8 s for High-Fast. Given these large differences between all but the intermediate conditions, we expected the optimal policy would be learned quickly by most participants.

**Fig. 1:**
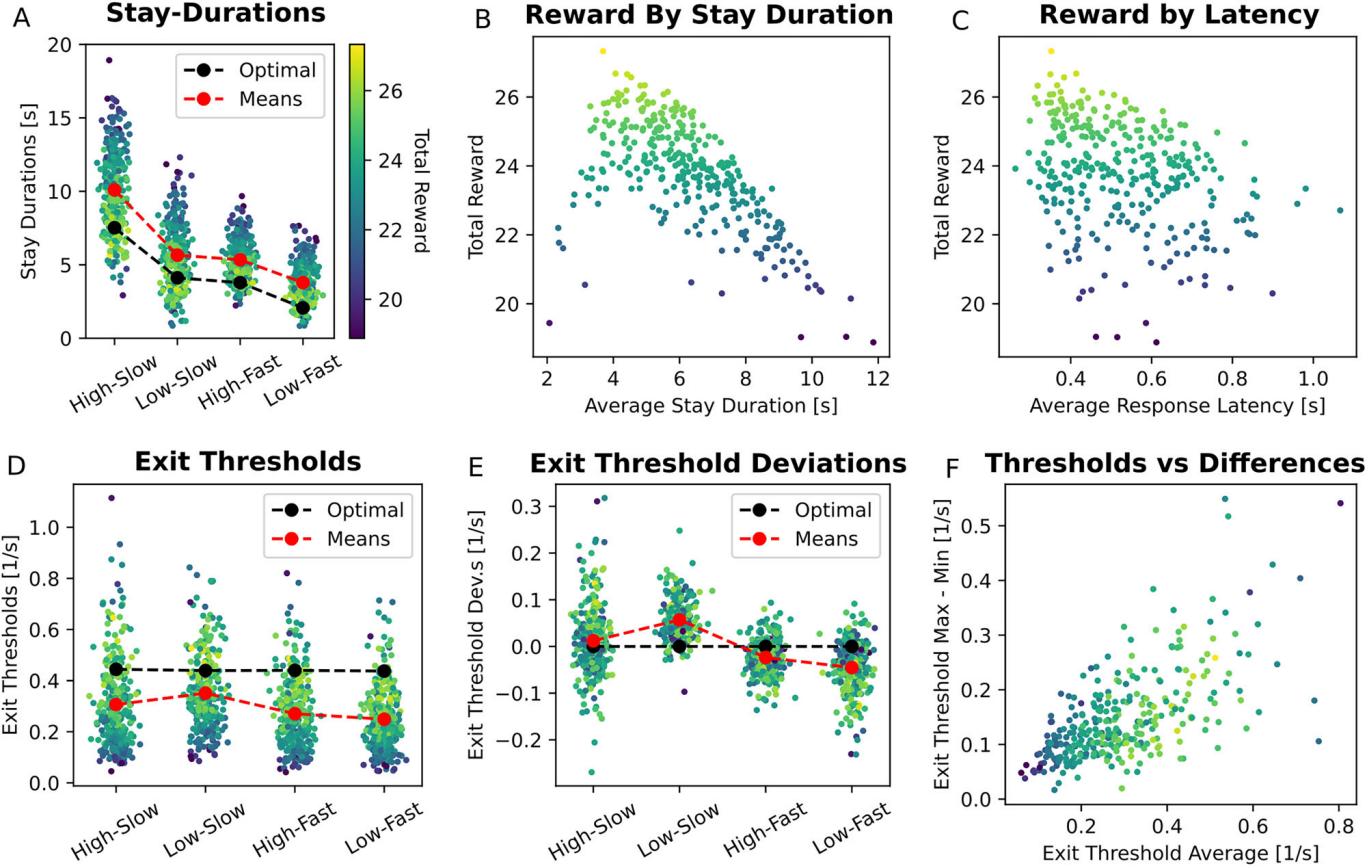
Summary of participant performance. **A** Average stay durations (over trials) for each task condition are plotted in a colour scale reflecting total reward obtained over the course of the experiment (lighter colours indicate higher reward, same colouring across panels). Participants stayed for longer than they should in all conditions, but recovered the rank ordering correctly (as a group). **B** Reward was strongly, but not entirely, determined by average stay durations. In particular, it was reduced by overstaying, which most participants exhibited, but also by under-staying, making the relationship nonlinear. **C** Response latency was a second significant determinant of total reward, and was not related to total stay durations (see main text). **D** Participants’ exit thresholds, which show differential overstaying by condition, with the least overstaying in the Low-Slow condition and the most in the Low-Fast. **E** Exit threshold deviations (from by-participant grand means) for each condition. Having a higher exit threshold deviation is generally better (less suboptimal). A minority of participants were in the vicinity of optimal performance, or even under-stayed, and for these participants, this higher-is-better interpretation will not always be correct. Participants can be seen to perform most suboptimally in the Low-Fast condition, followed by the High-Fast, High-Slow, and lastly, Low-Slow condition. **F** Participants with larger differences between their highest and lowest exit threshold, i.e., who had *less* consistent exit thresholds, had higher average exit thresholds and performed better on the task.

Participant behaviour indeed differed across conditions in accordance with normative predictions (Fig. 1A). Group means differed significantly between pairs of conditions (two-tailed paired t-tests; *t*_(316)_ = 22.9, 22.2, 14.8; *p* = 3.8e-85, 2.5e-2, 5.4e-43 for the three adjacent ordered comparisons starting with High-Slow vs. Low-Slow; *d* = 1.8, 0.2, and 0.9, respectively). As expected, the two middle conditions which had optimal stay durations within ~300 ms of one another had similar stay durations. Importantly, compared to optimal behaviour, most participants exhibited overstaying behaviour (Fig. 1A), and this had a substantial impact on total reward in the task (Fig. 1B). Group mean stay durations were substantially longer than those of an optimal agent, with mean overstaying of 2.6 s, 1.6 s, 1.6 s, and 1.7 s for the High-Slow, Low-Slow, High-Fast and Low-Fast conditions (two-tailed one sample t-tests; *t*_(316)_ = 16.9, 13.0, 20.5, and 23.8; *p* = 6.6e-46, 2.8e-31, 6.3e-60, and 2.5e-72; *d* = 1.0, 0.7, 1.2, and 1.4). Moreover, a second primary determinant of reward in the task was response latency (Fig. 1C), which was correlated with total reward (Pearson’s *r* = −0.33, *p* = 1.6e-9), but was unrelated to average stay durations (Pearson’s *r* = −0.02, *p* = 0.8).

Similar to stay durations, participant exit thresholds (their reward rates at exit time) were roughly half to three-quarters of the optimal values (Fig. 1D) (optimal threshold: 0.44 L/s; average thresholds: 0.30, 0,35, 0.27, 0.25 L/s for the four conditions, ordered as above). Overstaying behaviour, measured as exit threshold differences, was different across task conditions (two-sided paired sample t-test, *t*_(316)_ = −9.7, 2.8, 6.4; *p* = 8.5e-21, 1.3e-111, 3.5e-10; *d* = −0.77, 2.21, 0.50, ordered as above). This means that participants overstayed more in some conditions than others in terms of their exit thresholds. Specifically, participants performed more optimally in the two slow decay rate conditions, compared to the worst (Low-Fast) condition (compare red to black in Fig. 1D). This tendency cannot be explained by a fixed overstay duration pattern, as optimal stay durations were similar in the intermediate-quality conditions (see above).

Examining individual participant exit thresholds in more detail, we computed each participant’s variation around their own exit threshold baseline (Fig. 1E). This confirmed that *individual* participants tended to perform most suboptimally in the fast decay rate conditions, and most optimally on the Low-Slow condition. Many participants also overstayed in the High-Slow (best) condition more than in the Low-Slow (second best) condition. Participant suboptimality was therefore most pronounced in the fast decay rate conditions, over and above the (average) exit thresholds. One possible explanation for this may be that participants are less capable of approximating an internally defined exit threshold in the fast decay rate conditions (because of the higher rate of reward change, and perhaps an associated response lag) than in the slow decay rate conditions, but this would not explain the difference between High-Slow and Low-Slow suboptimality. Notably, the differences between participants’ highest and lowest exit thresholds were also significantly correlated with their average exit thresholds (Pearson’s *r* = 0.65, *p* = 1.3e-38; Fig. 1F), suggesting that the magnitude of this suboptimality difference is significantly related to the flattening reward rate curves at increasing stay durations. Taken together, better performing (more optimal) participants had *less* consistent exit thresholds than poorer performing ones.

### Individual differences in suboptimality

**Fig. 2:**
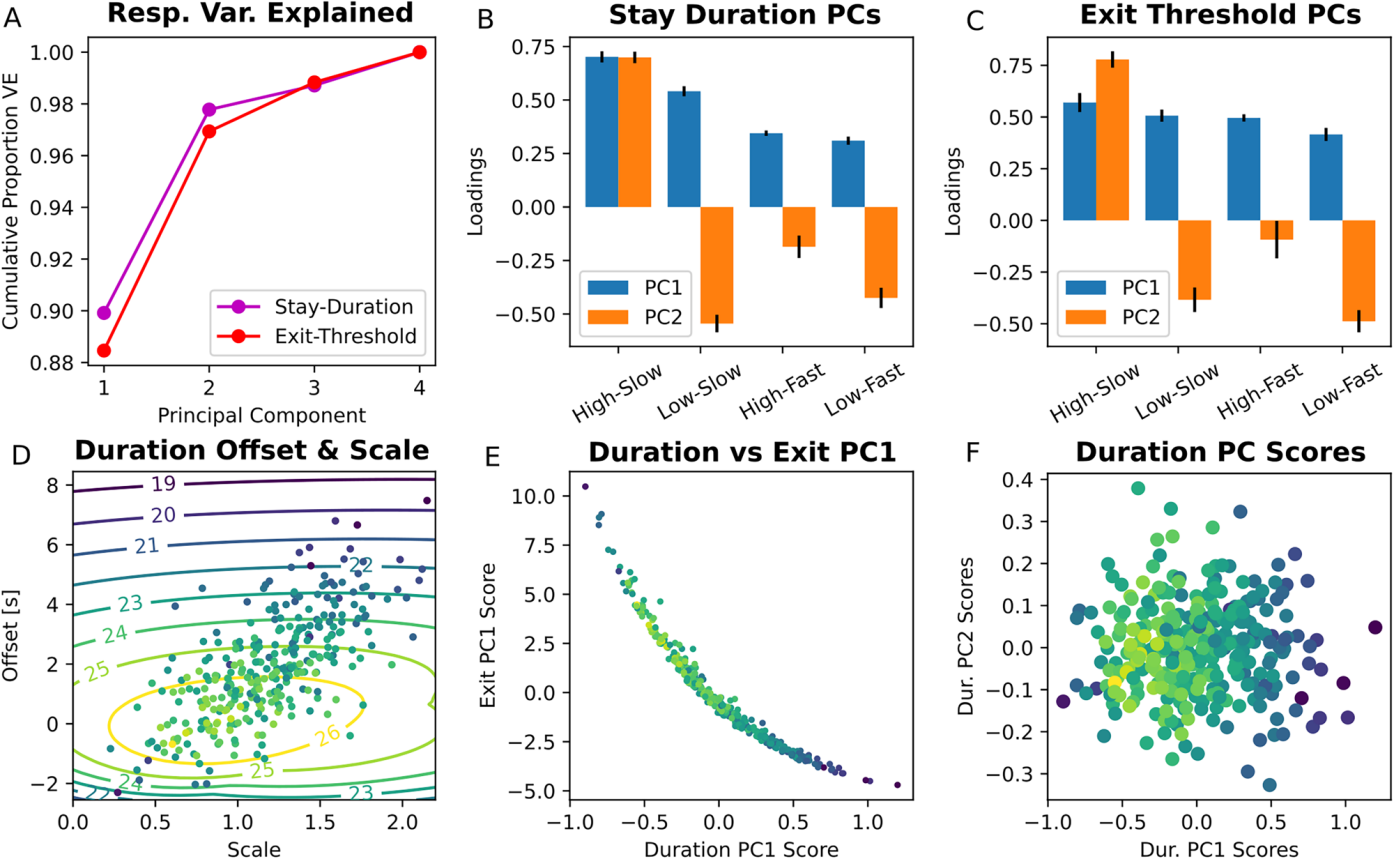
Participants mainly varied in their baseline stay durations, scaling of condition differences, and their preference for staying in the high-slow condition. **A** In both duration-space and exit threshold-space, the first PC explained roughly 90% of the variance, and the second PC explained most of the remaining variance. **B** PCs for participant average policies. The top PC combines baseline shifting and condition-difference scaling. The second reflects preference for the high-slow condition. Error bars show standard errors, estimated via jackknife resampling. **C** PCs for participant average exit thresholds by condition. Note the approximately flat structure of the first PC. Converting uniform exit threshold variation into stay durations produces the shifted and scaled variability seen in PC1 of (**B**). Parallel structure across (**B**) and (**C**) indicates that apparent condition preference structure in the data is robust, and is not an artefact of measuring either time-domain data or exit threshold data in particular. **D** Duration PC1 involves both a total stay duration offset (y-axis) and an exaggeration of condition differences (a scaling component, x-axis). The best performing participants had offsets near 0 and scale factors near 1 (normalised to differentiation under the optimal policy). Reward contours are plotted, showing that scaling had little effect on performance, whereas offset had a strong effect. **E** Duration and exit PCs scores are highly correlated, but exit PC1 scores saturate, showing a floor effect, because they are bounded by zero as stay durations increase. **F** The first stay duration PC was highly correlated with reward, but not the second PC.

To further examine the differences in foraging policies across participants, we performed PCAs on stay durations and exit thresholds. The first two principal components explained 90%, and 8% of the variance in stay durations across participants, respectively, and we obtained similar results for exit threshold PCs (Fig. 2A). The first PC (PC1) represented a combination of average stay duration shifting and scaling (Fig. 2B, C). Specifically, PC1 loaded positively on all stay durations, indicating baseline (or average) offsets between participants, but increased more for the ‘better’ two conditions than the ‘worse’ ones, indicating scaling or exaggeration of the between-condition stay durations. We found this to be a consequence of transforming approximately uniform exit threshold variations into duration space. In particular, the exit threshold variation (PC1 in Fig. 2C) implied greater stay duration variability in the slow decay conditions (PC1 in Fig. 2B) and vice versa.

Overstaying behaviour therefore had two components: a baseline stay duration offset (relative to the optimal set of stay durations), and a condition-difference exaggeration component. Examining this decomposition further, we computed participant’s offset components by taking mean stay durations, and computed their scale components by projecting mean-subtracted stay durations onto a mean-subtracted, unit-normed transform of the optimal policy. These components were highly correlated (Pearson’s *r* = 0.69, *p* = 7.0e-47), suggesting that participants that overstayed more, tended to exaggerate condition differences as well (Fig. 2D).

In contrast to PC1, the second PCs in both stay duration and exit threshold spaces measured a tendency to stay in the best (High-Slow) condition over all others (Fig. 2B, C). Therefore, systematic variation between participants was dominated by differences in their baseline overstaying behaviour and scaling (PC1: overstay/scaling) and their expressed preferences for the single best patch in the task (PC2: High-Slow preference). In subsequent analyses, where possible, we chose to primarily analyse participant stay behaviour in stay duration space rather than exit threshold space, since the two were strongly related, and exit reward-rates asymptoted to zero as stay durations increased (Fig. 2E). Both PC1 and PC2 remained consistent over the course of the experiment (see Supplementary Material).

Differences in overstaying/scaling (PC1) were strongly related to total reward earned in the task. In line with the strong impacts of average policy stay durations across conditions, participants with lower PC1 scores tended to earn more reward (Pearson’s *r* = −0.63, *p* = 1.1e-35) (Fig. 2F). When breaking PC1 down to baseline overstaying and scaling, the correlation between PC1 and reward was largely driven by a strong relationship between overstaying and reward (Pearson’s *r* = −0.63, *p* = 4.9e-36), whereas there was no association between scaling and reward, independent of the relationship with overstaying (when scaling residualised by baseline overstaying, Pearson’s *r* = −0.01, *p* = 0.89). Similarly, the orthogonal aspect of preference for the best task condition (PC2) did not meaningfully impact participants’ earned rewards (Pearson’s *r* = 0.02; *p* = 0.73).

### Age-related differences in foraging

We investigated the age-related differences in task performance, while accounting for individual differences in cognitive abilities and in motivational and depressive symptoms. Cognitive abilities included planning ability and processing speed, measured with accuracy and response time in the Tower of London task and a simple reaction time task. Motivational and depressive symptoms were measured with the AES and PHQ-9, respectively. These variables are summarised for each group in Table 1.

**Table 1:** Summary of motivational and depressive symptoms as well as cognitive performance across age groups

Variable	Young adults	Older adults	*t*-statistic	*p*-value*
Age	30.83 ± 0.41	69.16 ± 0.41		
Apathy Evaluation Scale	35.44 ± 0.67	32.45 ± 0.87	2.71	0.007
Patient Health Questionnaire-9	4.90 ± 0.31	2.21 ± 0.30	6.32	1.26e-09
Tower of London accuracy	17.79 ± 0.20	15.70 ± 0.35	5.16	8.10e-07
Tower of London reaction time for correct responses	6.052 ± 0.116	7.242 ± 0.147	−6.33	1.69e-09
Median reaction time in reaction time task	0.373 ± 0.003	0.430 ± 0.006	−7.61	4.84e-12

Values are mean ± standard error of the mean. Reaction time values are shown in seconds. *Uncorrected *p*-values.

**Fig. 3:**
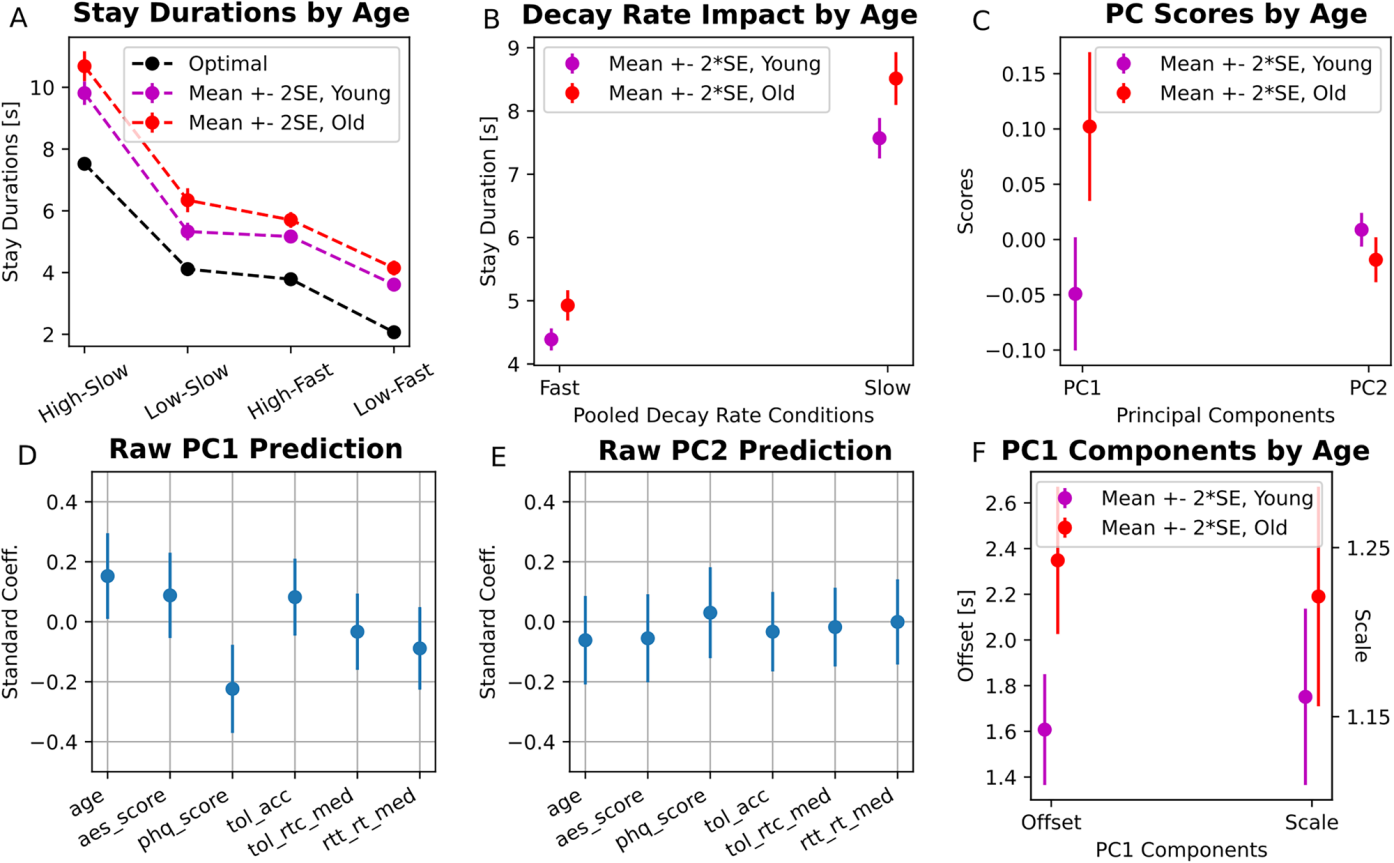
Effect of age on foraging behaviour. **A** Average stay durations (over trials and participants) for each task condition are plotted separately for young (magenta) and older adults (red), together with optimal performance (black). **B** Average stay durations plotted separately for the fast and slow decay rate conditions, and separately for young and older adults. Error bars indicate two standard errors. **C** Same as (**B**) but for the principal component (PC) scores for the first (PC1) and second PC (PC2). **D** Regression coefficients for the linear model predicting participant overstaying/scaling (stay duration PC1). Age, Apathy Evaluation Scale score (aes_score), Patient Health Questionnaire-9 (phq_score), Tower of London accuracy (tol_acc), median reaction time for correct responses in Tower of London (tol_rtc_med), and median reaction times in reaction time task (rtt_rt_med) were all included as predictors in the model. Error bars indicate 2 standard errors. **E** Same as (**D**), bur for the association with a preference for best condition (stay duration PC2). **F** The two components of PC1, namely offset and scale, plotted separately for young and older adults. Error bars indicate 2 standard errors.

Overall, older adults stayed consistently longer compared to young adults across conditions (Fig. 3A). As participants were overall less optimal in the fast decay conditions (see Fig. 1D), we examined whether this suboptimality was exaggerated in older adults (Fig. 3B). A linear mixed effect model confirmed that older adults stayed more overall (*β* = 0.075, SE = 0.018, *t*_(15326)_ = 4.184, *p* = 2.9e-5), and showed a small but significant decay rate × age interaction (*β* = 0.012, SE = 0.006, *t*_(15326)_ = 1.98, *p* = 0.048). The interaction suggests that older adults overstayed more, in the slow decay rate conditions than would be expected by age or decay-rate alone.

To explain differences in stay durations between groups, we compared group behaviour in terms of overstaying/scaling (PC1) and preference for best condition (PC2) (Fig. 3C). Compared to young adults, older adults showed a significant increase in PC1 (*t*_(309)_ = 3.52, *p* = 0.001) but a reduction in PC2 (*t*_(309)_ = −2.354, *p* = 0.019). The difference in PC1 (*β* = 0.152, SE = 0.072, *t*_(272)_ = 2.130, *p* = 0.03) but not in PC2 (*β* = 0.0615, SE = 0.074, *t*_(272)_ = - 0.834, *p* = 0.41) remained significant after accounting for cognitive performance, motivational and depressive symptoms (Fig. 3D, E). The consistent group difference in PC1 was driven by a strong difference in offset (*t*_(309)_ = 3.638, *p* = 3.4e-4) and not by a difference in scaling (*t*_(309)_ = 1.238, *p* = 0.220) (Fig. 3F). These results suggest older adults showed a greater tendency to overstay but with a similar scaling to each reward condition.

**Fig. 4:**
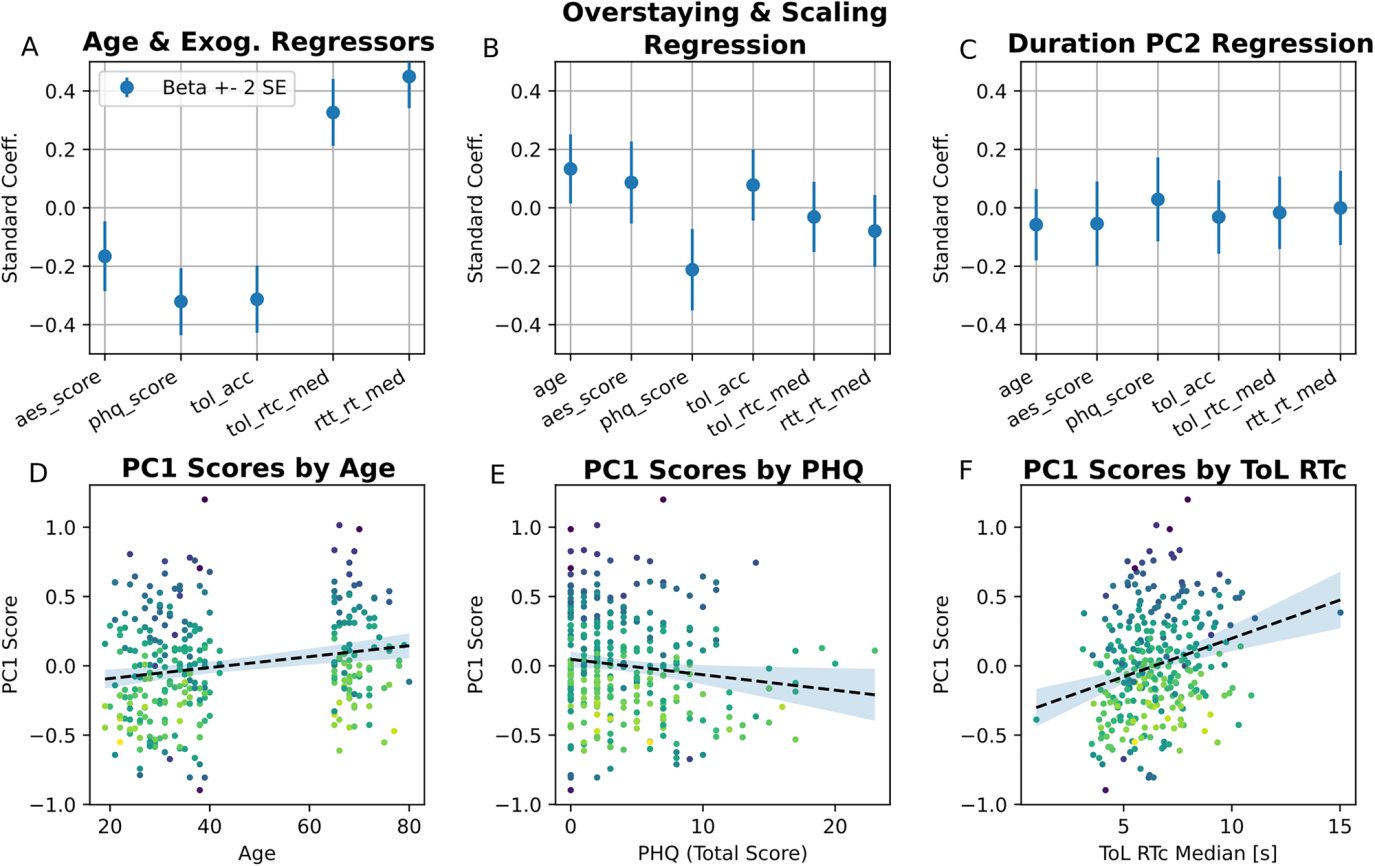
Unique contribution of age, cognitive performance, motivational and depressive symptoms to foraging behaviour. **A** Regression coefficients predicting age, according to Apathy Evaluation Scale score (aes_score), Patient Health Questionnaire-9 (phq_score), Tower of London accuracy (tol_acc), median reaction time for correct responses in Tower of London (tol_rtc_med), and median reaction times in reaction time task (rtt_rt_med). Error bars indicate 2 standard errors. **B** Regression coefficients for linear models predicting participant overstaying/scaling (stay duration PC1). **C** Same as (**B**), bur for the association with a preference for best condition (stay duration PC2). **D** PC1 scores by age, with a linear regression line and 95% confidence interval. **E** Same as (**D**), but for PC1 scores by PHQ. **F** Same as (**D**), but for PC1 scores by Tower of London median correct reaction time (RTc).

Lastly, we explored the *unique* contributions of each of the variables: age, cognition, motivational and depressive symptoms on foraging behaviour. To this end, we residualised our task-exogenous variables by age, as age was significantly associated with all variables (Fig. 4A). These variables were then entered into two regression analyses, predicting PC1 and PC2. In the first regression model (Fig. 4B), age and PHQ-9 scores showed significant associations with PC1 (*β* = 0.13, −0.21; SE = 0.06, 0.07; *t*_(274)_ = 2.3, −3.50; *p* = 0.02, 0.003, for age and PHQ-9, respectively). By contrast, none of the variables was associated with PC2 in the second regression model (Fig. 4C). Post hoc zero-order correlations showed there was a positive association between PC1 and age (*r* = 0.20, *p* = 4.3e-4, uncorrected; Fig. 4D), a negative association with PHQ-9 (*r* = −0.13, *p* = 0.025, uncorrected; Fig. 4E). For completeness, we also illustrate the positive association between PC1 and response times in the Tower of London task (Fig. 4F), which was abolished when residualising by age.

## Discussion

The present study investigated the age-related differences in suboptimality in foraging behaviour. We found that one of the main forms of suboptimality in foraging, namely overstaying, was increased with age. Other forms of suboptimality in our task, namely increased overstaying in fast decay rate, condition-specific exaggeration of stay duration, or preference for the best condition, were all not consistently associated with age. An additional analysis revealed that individuals with lower depressive symptoms also independently showed exaggerated overstaying. Together, our results suggest that older adults show exaggerated overstaying, consistent with the exploitative mental mode hypothesis for ageing^12^.

As reported in previous research, we found that most participants consistently overstayed in a patch, beyond what is predicted by normative models, such as the MVT^16^. Several cognitive and motor mechanisms have been suggested to explain overstaying tendency. First, temporal discounting^11^ can make individuals overstay because leaving one source of reward incurs a cost in the form of a time delay before the next source of reward becomes available. This time delay spent on ‘travelling’ reduces the value of the future reward^11^. Second, sensitivity to costs or effort in the task may contribute to overstaying, specifically in relation to costs incurred during travel to a different patch or while extracting reward within a patch (i.e., harvesting). Although we did not directly manipulate the effort involved in harvesting or travelling^17^, individuals may perceive the effort of harvesting by holding down the button and monitoring the filling milk bucket differently. Those who are less sensitive to this effort would exhibit more overstaying behaviour. Third, foragers need to infer the structure of their environment and use the uncertainty about this inferred structure to adjust their strategies^8^. This means that future reward value is discounted by uncertainty about the environment, leading to overstaying behaviour^8^. Fourth, overstaying can be explained by perseveration—whether cognitive or motor^18,19^—that is, the inappropriate, or in this case suboptimal, prolongation of behaviours (sometimes termed ‘continuous perseveration’^20^).

Adding to these contributors to suboptimality in foraging, we found two distinct behavioural patterns that deviate from optimality: 1) a strong association between baseline overstaying and condition-specific exaggeration of overstaying; 2) a condition-specific deviation of exit thresholds from optimality. First, we found that the tendency to overstay (‘offset’) was highly correlated with condition-specific exaggeration of stay durations (‘scale’). In other words, participants who tended to overstay for longer, were also more sensitive to changes in the reward conditions. This suggests that overstaying tendency cannot be simply explained by a reduced attention to the task or general slowness, which was indeed not associated with this behavioural pattern in our study (no associations with reaction time). Instead, people who tend to stay longer may place a greater subjective value on extracting reward from each condition, which also makes them more sensitive to differences between conditions. That is, people may suboptimally perceive (over)staying as persistence in the task, and hence those who are more engaged and reward-driven may counterproductively overstay in each patch. This interpretation would also explain the negative association between overstaying/scaling and depressive symptoms (see below).

Second, we found that the extent of suboptimality differed between reward conditions. Specifically, our task allowed us to examine the differential effect of initial reward rate vs. reward decay rate. While overstaying was similar in terms of stay durations across conditions, people overstayed more in fast relative to the slow decay rate conditions in terms of exit threshold. Initial reward rate, by contrast, did not affect the degree of suboptimality in the task. These findings are at odds with theories such the MVT, which compute the optimal exit thresholds as a function of current reward rate relative to average environmental reward, without considering the specific factors that contribute to the instantaneous reward rate. Our findings suggest that other contextual or cognitive mechanisms might contribute to suboptimality in foraging. For example, the larger deviation from optimality in fast decay rate can be due to a limitation in cognitive flexibility or suggest that individuals favour stability in their reward-seeking behaviours, even in environments that would benefit from more agile adjustments.

Consistent with the exploitative mental mode hypothesis, older adults demonstrated a greater tendency to overstay in a patch compared to younger adults. Such a tendency would suggest reduced, rather than heightened sensitivity to opportunity cost of time^7,13^. These findings could not be explained by differences in basic cognitive performance, as this was accounted for in our analyses. We note, however, that although we accounted for individual differences in processing speed and executive function, differences in other cognitive domains could potentially explain the exaggerated overstaying in our study, such as age-related increase in perseveration^21–23^. Moreover, older adults overstayed less in the fast decay rate condition, compared to young adults, suggesting intact responsiveness to the diminishing reward (see more below). Further, although persistently increased uncertainty could lead to overstaying in older adults^8^, potentially due to impaired learning of task structure and/or reduced cognitive control^12^, our task was specifically designed to be simple and to involve minimal learning, as evidenced by stable split-half performance (see Fig. S3).

Instead, overstaying in older adults could reflect a shift towards exploitation-, rather than exploration-based behaviour. Behaviourally, several explanations have been proposed for this shift, including a greater reliance on prior knowledge^12,24^ which increases the risk and diminishes the value of exploratory options. Alternatively, positivity bias with age^25,26^ has also been proposed to bias behaviour towards an exploitation of known, positively valenced options. Neurally, the degradation in noradrenergic circuitry has been proposed to lead to less flexible shifting from exploitative behaviour^12^.

Interestingly, although overstaying and sensitivity to reward conditions in our task were highly correlated, older adults behaved similarly to young adults in terms of condition-specific adaptation of stay durations. This intact sensitivity to reward decay speaks to a large body of literature looking at reward processing in ageing^27^. Previous research has shown that reward processing changes in the brain across the lifespan^28–30^, but behaviourally, there are no clear age-related changes in reward sensitivity^7,27,31^. The lack of a consistent behavioural change could be related to the inconsistent age-related changes in dopamine^31^.

Dopamine has been suggested to control responsiveness to reward in the decision to switch^10,32–34^. Specifically, dopamine drives switching behaviour^32,34^, particularly in low-reward or ‘poor’ environments^10^, and it has been suggested that tonic levels of dopamine reflect average reward rate^33^. Thus, a simplistic interpretation of our finding could be that overstaying behaviour in older adults is related to lower tonic dopamine levels. However, this interpretation would need to be reconciled with the finding of intact sensitivity to reward decay rate in older adults, which is in turn thought to be more related to phasic dopamine levels^33^. Future studies could more specifically test these hypotheses, for example in groups of patients with Parkinson’s disease where tonic dopamine is primarily affected^10^.

We found a negative association between overstaying/scaling and depressive symptoms, as measured by the PHQ-9. This association indicates that individuals with more depressive symptoms tended to stay in a patch for a shorter duration. This finding is consistent with our interpretation that overstaying in the task could be seen as effortful or as persistence in the task. On this account, more depressed individuals experience lower subjective value for extracting reward in each patch, leading them to switch earlier and have lower exit thresholds than those with less depressive symptoms. This result is also consistent with theories of lower average reward expectations in depression^2,35^. Moreover, a reduced scaling or reward sensitivity (tightly linked to overstaying in our task) aligns with existing research showing diminished reward sensitivity in individuals with depression^36^.

The lack of an independent association between foraging and apathy, as measured by the AES, is in contrast with previous research linking apathy to: the opportunity cost of time^37^; to temporal discounting and impulsivity^38–41^; to perseveration^42,43^; and to an inflated sensitivity to effort^15,44–47^ (but see ref. ^48^). Interestingly, another recent study found no significant relationship between task parameters in an effort foraging task and apathy, although measured using a different apathy scale^17^. Moreover, a different study reported only a weak association between AES and choice bias in an effort-reward decision-making task, but no association between AES and effort or reward sensitivity^49^. Still another study reported no association between AES and behavioural parameters in an effort-reward decision-making task^48^. These studies raise the possibility that in the general population, where changes in apathy scores are more subtle than in clinical populations, and where the meaning and validation of clinical scales such as the AES are less established, associations with task parameters can be weak.

The strengths of our study include the use of a new time-constrained foraging task, together with cognitive tasks looking at processing speed and executive function. When measuring more complex behaviours with new tasks, and especially when testing differences between populations, such as older adults or patient groups, it is crucial to consider new tasks in conjunction with well-established measures of robustly identified cognitive traits, as we did here. However, our study has several notable limitations. First, the study was cross-sectional, and our results could not assess longitudinal changes with age. Moreover, we used a simple foraging task that would involve minimal learning and could potentially be applied to individuals with varying degrees of cognitive impairments. However, this meant that the task was more limited in what it could measure, as for example only one environment was included, and we did not manipulate effort or background reward rate as has been elegantly done before^10,17^. Lastly, this study was largely descriptive, and a more mechanistic account of task-related behavioural differences would, of course, be preferable.

In conclusion, our study finds that suboptimal foraging behaviour arises from multiple behavioural factors, which are not consistent with fixed exit threshold-based computations. In contrast to predictions from normative theories, overstaying was strongly associated with adaptation to reward conditions. Despite this association, older adults showed a selective increase in overstaying without a corresponding increase in reward-based adaptation. This effect could not be accounted for by slower processing speed, differences in executive function, or variations in motivational or depressive symptoms. Together, the results suggest that older adults exhibit an exaggerated tendency to overstay, which may reflect a shift towards more exploitative decision-making. Our study highlights the need for more comprehensive theoretical frameworks and computational models that can explain systematic variations in foraging behaviour, particularly across the lifespan.

## Methods

### Participants

We recruited participants through Prolific (https://www.prolific.co/). Screening criteria included age of 18–45 years for the young adult group and 65+ years for the older adult group, verified identity on Prolific, no chronic condition/illness, and no ongoing mental health/illness/condition. Participants also had to pass a colour-blind test. All participants were asked to read the study information pages and to confirm they are eligible to participate and their consent to participate in the experiment. Participants were paid £4.50 (~ £9 an hour rate), which included a fixed £1 bonus (see below). The study was approved by the Tel Aviv University ethics committee (ref: 0003132).

Due to the limited availability of eligible older adults on Prolific at the time of data collection, we opted for a 2:1 recruitment ratio of younger to older participants, while ensuring sufficient power to detect the expected age-related effects. Power analysis showed that with a 2:1 young-to-older adult group ratio, and a sample size of 230 young adults and 115 older adults, the study would have >95% power to detect significant associations between foraging performance and the variables of interest: age, depression and apathy in two regression analyses (see below), while controlling for the three cognitive variables (see below), with *f*2 = 0.15, *ɑ* = 0.05/2. We therefore recruited 235 young adult participants (96 female) with a mean age = 30.81 years and SD = 5.79 years (age range 19–42 years) and 120 older adult participants (54 female) with a mean age = 69.26 years and SD = 3.81 years (age range 65–80 years). The data of five participants was not considered due to a technical error in data collection leaving a total of *n* = 350.

### Experimental procedures

Participants performed three behavioural tasks and two questionnaires. The tasks examined foraging behaviour (milkman task), basic processing speed (choice reaction time task), and executive function or planning abilities (Tower of London task).

#### Milkman task

**Fig. 5:**
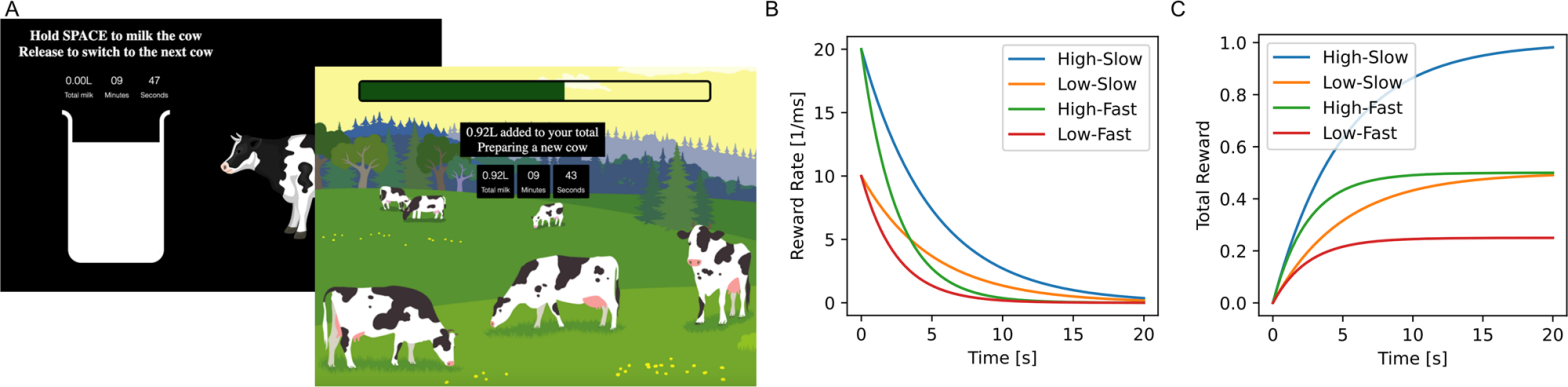
Foraging in the milkman task. **A** In the task, participants had 10 min to maximise the amount of milk they collect. In the main milking screen, participants held the spacebar key to fill a bucket with milk. Animated milk filling the bucket showed participants the milk accumulation rate, and participants chose when to release the spacebar key in order to switch to the next cow. Switching cows (‘travel’) resulted in a fixed (4 s) time cost. Milk accumulation rate followed an exponential function, and there were four types of cows (uncued, pseudo-random differences, observable only via reward accumulation), each combining one of two initial milk accumulation (reward) rate and one of two reward decay rate parameters. **B** Instantaneous reward-rates over time by condition. **C** Total reward accumulation by time for each condition.

Inspired by Le Heron et al.^10^, we developed a task that operationalises the decision to switch in a time constrained experiment (Fig. 5A). Participants were asked to collect as much milk as possible in a 10-min experiment. To incentivise them, they were informed that only those ranked in the top third in terms of total amount of milk collected would receive a bonus payment of £1.

To milk a cow, participants held down the spacebar key while a bucket, representing the accumulating milk, was displayed on the screen (‘milking screen’). They were informed that as milking continued, it would become increasingly difficult to obtain milk from a cow. In reality, the milk accumulation rate followed an exponential function (Fig. 5B) of the form:

$${N}_{\left(t\right)}={N}_{0}{e}^{-\lambda t}$$

Where ${N}_{\left(t\right)}$ is milk accumulation rate at time *t*, and ${N}_{0}$ is the initial accumulation rate and $\lambda$ is the milk accumulation decay rate. The total milk accumulated (Fig. 5C), as shown in the bucket, reflected the closed-form integral of this function. ${N}_{0}$ was taken from the set {0.01, 0.02} and *λ* from the set {2e^−4^, 4e^−4^}, creating a 2 × 2 design and four cow ‘types’ or conditions with high/low initial reward rate and fast/slow reward decay rate: High-Slow, High-Fast, Low-Slow, Low-Fast. These specific values were chosen, so as to have one best condition (High-Slow), one worst condition (Low-Fast) and two intermediate conditions which would be similar in optimal stay durations (High-Fase and Low-Slow). Conditions were interleaved and pseudorandomly presented, such that each cycle of four consecutive trials contained each condition.

After releasing the spacebar, participants saw a ‘travel screen’, where they were told the next cow was being prepared for milking. Travel time was fixed at 4 s, and a progress bar was displayed showing the remaining time for the travel screen. On the travel screen, participants could see how much milk was added to their total. Participants were discouraged from pressing the spacebar key during the travel screen, and if premature pressing occurred, they received a warning on the screen which appeared for 2 s. Throughout the experiment (during both milking and travel screen), time remaining was displayed as a countdown timer, as well as the total milk acquired so far. The experiment began with a 1-min practice to familiarise participants with the task.

#### Self-report scales

To account for the relationship between foraging and apathy and depression symptoms, participants completed two self-report scales. First, Apathy Evaluation Scale (AES)^50^ was used to examine individual differences in apathy symptoms. Second, depressive symptoms were assessed using the nine-item Patient Health Questionnaire (PHQ-9)^51^. We included two attention check items per questionnaire in the format of nonsensical or ‘infrequency’ items^52^.

#### Control cognitive tasks. Choice reaction time task

In the choice reaction time task, two empty circles were displayed horizontally on the screen. After a pseudorandom interval of 1–5 s (drawn from a uniform distribution), one of the circles turned black, and participants were asked to press the left or right arrow key as fast as they can, if the left or right circles turned black, respectively. There were 50 trials overall, and the task took ~5 min. The measure of interest for each participant was median reaction time in ms, as overall accuracy (whether the button pressed corresponded to the circle turning black) was at ceiling (minimum 89% across individuals, with median accuracy of 100% across individuals).

#### Tower of London task

In the Tower of London task^53^, participants were instructed to move coloured discs, one by one, from a ‘start state’ depicted in one image, to match a ‘goal state’ depicted in another image. The discs are stacked vertically across three positions, with different configurations in each trial. During three practice trials, participants indicated the required number of moves to reach the goal state and then demonstrated these moves using a mouse. Only after correctly indicating and performing the moves did participants proceed to the experimental trials. In the 20 experimental trials, participants had 20 s to indicate the number of moves needed, without having to demonstrate the moves. The task was designed similarly to that in the Brief Assessment of Cognition in Schizophrenia^54^: It was terminated after five incorrect responses, and conversely, participants who were correct on all 20 trials completed two additional trials. The measure of interest was the total number of correct responses in the experimental trials.

### Exclusion criteria

We implemented several basic quality controls. Our data included some participants who did not engage with the task properly, including one that only completed several trials, waited approximately 8 mins (out of a total of 10 min), and a second that foraged in each patch for an average of 50–60 s. After excluding these two participants, we performed further exclusions by computing robust z-scores of stay durations, response latencies, stay duration Principal Component Analysis (PCA) scores (see below), and total reward, then removing participants falling outside of 3 robust SDs (defined as 1.48 times the median absolute deviation) on these measures (Fig. S1). Our exclusion criterion can be considered lenient, because it translates into an average stay duration threshold of over 10 s and an average latency threshold of over 1 s. Many of the participants who failed one of these tests also failed multiple of them (Fig. S2). Ultimately, our 3-SD outlier exclusion rule removed 31 participants from our foraging analyses, for a retention of 91% of the data, 317/350 participants for the main behavioural analyses.

For analysing the associations between task performance, age and motivation, we further excluded participants who either failed attention check questions on the AES or PHQ-9 or had implausible mean response times on the reaction time task. The implausible response times were also determined according to the 3-SD criterion, which translated into a threshold of approximately 1200 ms. Most participants failing this criterion had reaction times longer than 2.5 s, with some reaching 15 s. As a result, these set of analyses included 274 participants, comprising 78% of the original dataset.

### Statistical analyses

All analyses were performed in python version 3.11.7. In our analyses, we first computed the optimal policy for the task, by using the SciPy optimisation package, maximising reward with respect to stay durations. We defined a reward function mirroring that used in the task, which was parameterised by a fixed total experiment time of 10 min, an inter-patch travel time of 4 s, the experimental rates of return for each condition, and a fixed response latency of 650 ms across all patches (roughly the median participant latency, which has no effect on the optimal policy within reasonable bounds). Optimisation used an initial policy estimate with stay durations of 6 s, 5 s, 4.5 s and 3.5 s, and was robust to changes in this initialisation.

To examine the structure of foraging behaviour, we conducted PCA on stay durations and exit thresholds, where exit thresholds were the patch reward rate at the time people switched patches. The first two principal components (PC1 and PC2) were used in subsequent analyses. We decomposed PC1 into 1) baseline stay duration offset, measuring how much longer a participant stayed compared to optimal durations. This was calculated as the mean stay duration across all conditions; 2) condition-difference scaling, measuring how much a participant exaggerated the differences between conditions. This was calculated as the projection of mean-subtracted stay durations onto the normalised optimal policy. To assess task measure stability, we computed split-half reliability by comparing PCAs and exit deviations between first and second halves of the experiment. Correlations between halves were calculated using Pearson’s *r* for PC scores and rank correlations for exit deviations.

Finally, we investigated the relationships between foraging behaviour and age. To this end, we conducted two regression models with age predicting PC1 and PC2, and AES, PHQ-9, and cognitive performance in the two cognitive tasks as additional covariates. We also explored the unique contribution of each of these variables. To this end, we first residualised task exogenous variables against age, before entering them into two separate linear regression models, predicting PC1 and PC2. Statistical significance was assessed at *α* = 0.05, with specific p-values reported for key comparisons. Effect sizes were reported as Cohen’s d for group comparisons and Pearson’s *r* for correlations.

## Supplementary information

Wolpe_Supplementary Material

## Data Availability

All data used in the analyses are publicly available on the OSF platform at: https://osf.io/x6rqm.
