# Supplementary material for: Ageing is associated with exaggerated overstaying in foraging behaviour
Source: NPJ Aging. 2025 May 30;11(1):46. doi: 10.1038/s41514-025-00240-1 (PMC12125363; doi:10.1038/s41514-025-00240-1)
Supplement: Supplementary file 1 — Wolpe_Supplementary Material [file 41514_2025_240_MOESM1_ESM.pdf]

Supplementary Material

Supplementary Results

Consistency in task performance

To assess whether our task measures were stable over the course of the experiment, we examined split-halves of our data. Recomputing PCs using the initial and final data segments, we observed strong agreement over the course of the task for both PC1 (Fig. S3A) and PC2 (Fig. S3B) (cosine angles: 3.0 and 2.9 degrees for PC1 and PC2 initial vs. final). Similarly, the profile of exit deviations (computed as above) was conserved (Fig. S3C), although there was a notable increase in the relative optimality of participants' responses to the Low-Slow condition over the task (start vs end bars, Low-Slow group, Fig. S3C). In addition to PC stability, participants' initial and final scores were high correlated for both PC1 (Pearson's $r = 0.80$, $p = 1.5e\text{-}72$, Fig. S3D), and PC2 (Pearson's $r = 0.57$, $p = 4.4e\text{-}29$, Fig. S3E). Exit deviations were correlated at a range of values between about 0.3 and 0.6 (Fig. S3F). Taken together, these results therefore indicated that both group factor structure and individual participant scores reliably reflected suboptimality over the duration of the task.

Supplementary Figures

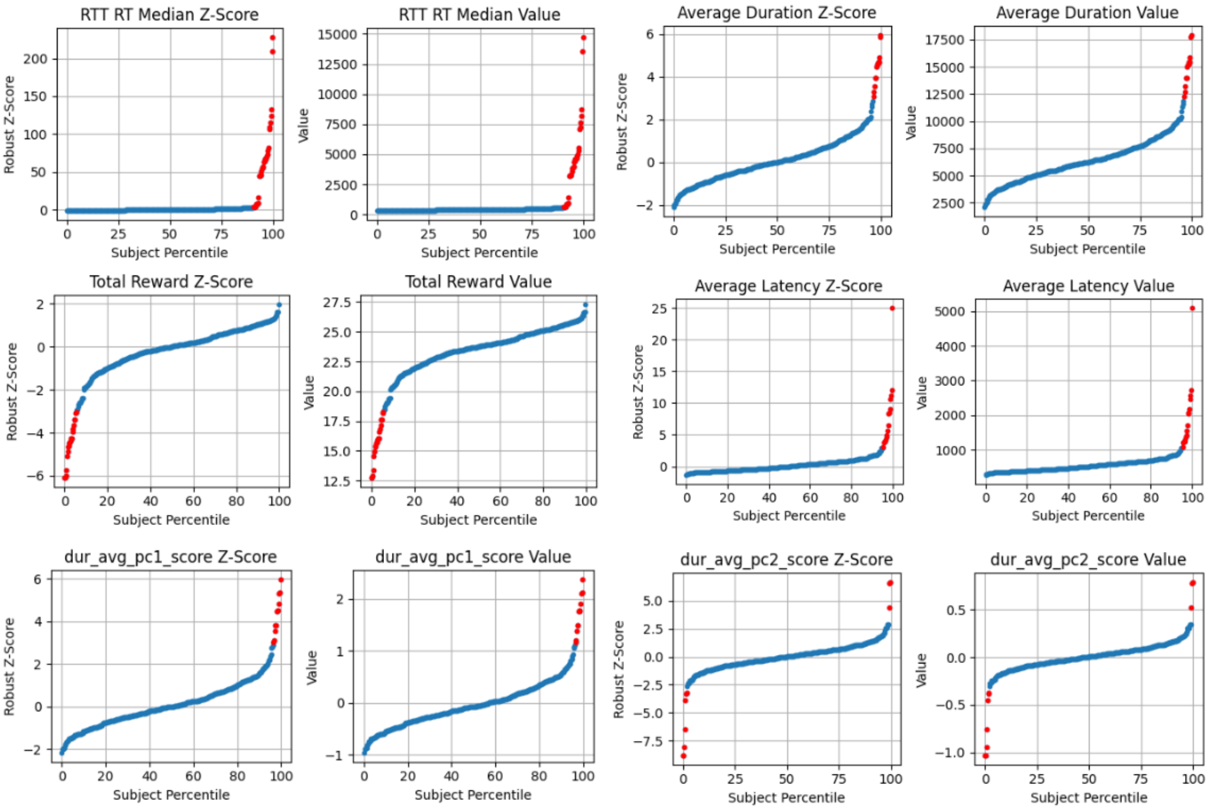

Figure S1. Participant exclusion across task variables. Participant reaction time, stay duration, latency, total reward, and Principal Component (PC) scores. Blue points are participants included in the analyses. Red points are outliers removed using a robust 3-SD cutoff for outlier detection.

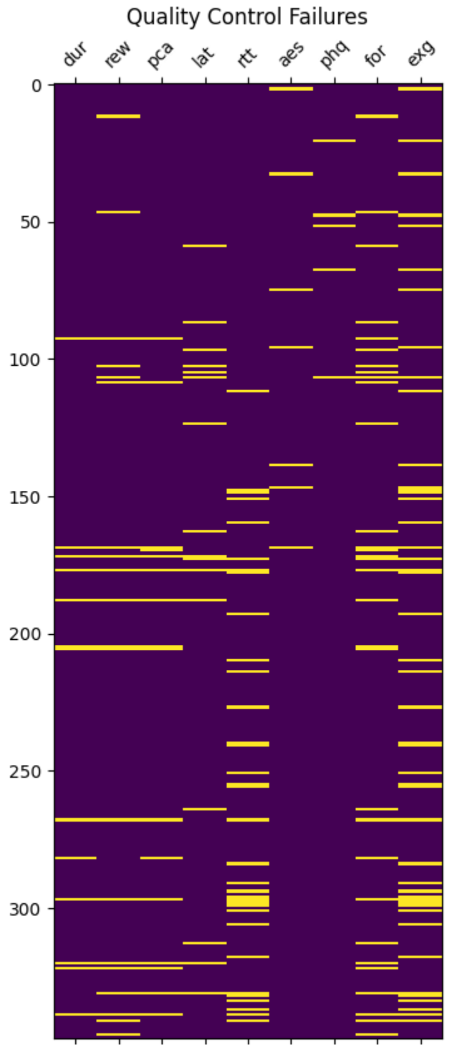

Figure S2. Participant quality control failures. Y-axis denotes participant number, x-axis denotes quality control metric, and a yellow bar indicates failure in a quality control check. Duration, reward, Principal Component Analysis (PCA) scores, and latency (first 4 columns) were used to remove participants for the foraging task analyses (PCs were subsequently recomputed). The reaction time task (RTT), Apathy Evaluation Scale (AES), and Patient Health Questionnaire-9 (PHQ) columns show participants who failed our task exogenous quality control, with most exclusions taking place because of implausibly high average reaction times in the reaction time task. The columns “for” and “exg” are aggregated exclusions from the foraging and exogenous task analyses.

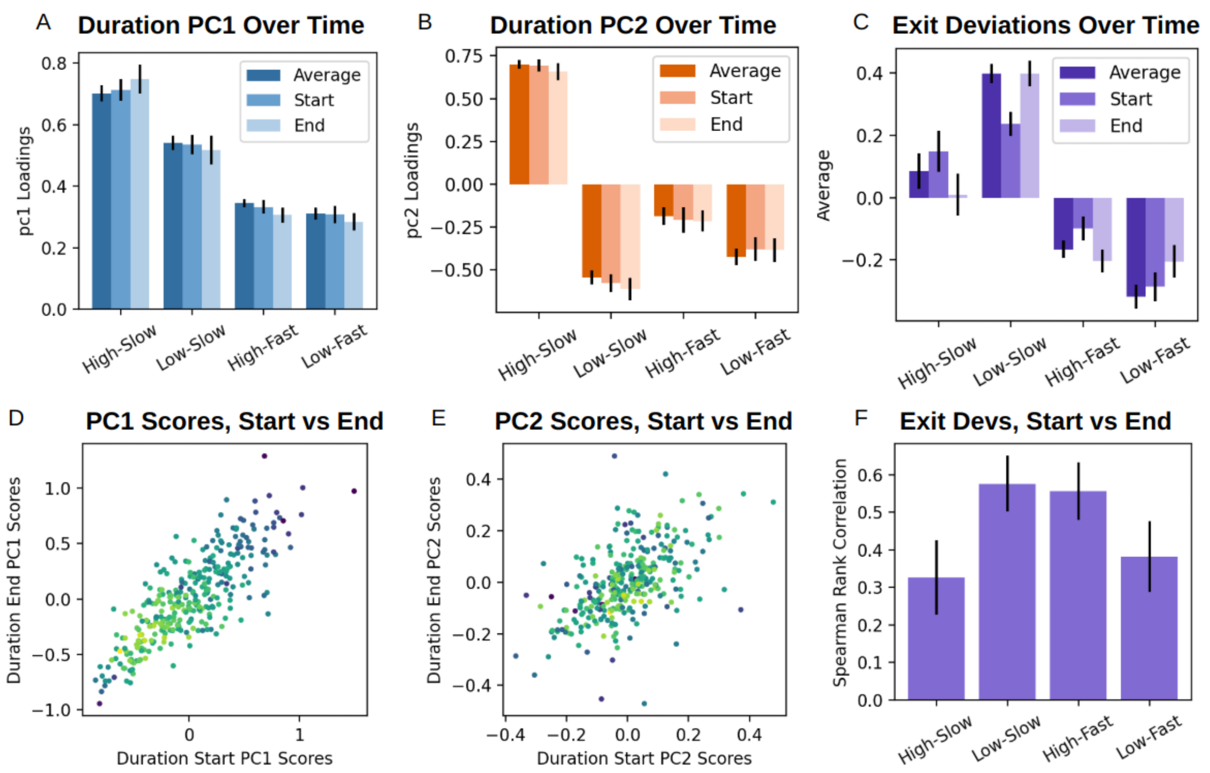

Figure S3. Stability of measures over experiment duration. (A-B) Principal components of participant stay durations, computed from the first and second halves of the task, were consistent with one another, and with those computed from the whole task. Error bars for loadings are 2 standard errors, computed via jackknife resampling. **(C)** Exit deviations over the course of the task were largely stable as well, although there was evidence that participants become systematically more suboptimal in the Low-Slow condition. Bars are means and error bars are +/- 2 SEM. **(D-E)** Scatterplots of participant PC1 (respectively, PC2) scores on the first and second halves of the experiment. **(F)** Rank correlations of participant exit deviations on the first and second half of the experiment.
